# Molecular Analysis of Antimicrobial Resistance among *Enterobacteriaceae* Isolated from Diarrhoeic Calves in Egypt

**DOI:** 10.3390/ani11061712

**Published:** 2021-06-08

**Authors:** Abdel-Moamen E. Meshref, Ibrahim E. Eldesoukey, Abdulaziz S. Alouffi, Saleh A. Alrashedi, Salama A. Osman, Ashraf M. Ahmed

**Affiliations:** 1 Department of Bacteriology, Mycology and Immunology, Faculty of Veterinary Medicine, Kafrelsheikh University, Kafrelsheikh 33516, Egypt; dr_moamen_meshref2002@yahoo.com (A.-M.E.M.); ashrafa5@yahoo.com (A.M.A.); 2 King Abdulaziz City for Science and Technology, Riyadh 11442, Saudi Arabia; 3 Central Laboratory at Al Watania Poultry Company, Riyadh 51441, Saudi Arabia; saalrash1@gmail.com; 4 Department of Animal Medicine and Infectious Diseases, Faculty of Veterinary Medicine, Kafrelsheikh University, Kafrelsheikh 33516, Egypt; salama2068@yahoo.com

**Keywords:** antimicrobial resistance, *Enterobacteriaceae*, calves, diarrhoea

## Abstract

**Simple Summary:**

Bacterial antimicrobial resistance is a serious global health challenge. This study investigated the occurrence of major antimicrobial resistance genes, including integrons, ß-lactamases, and florfenicol in *Enterobacteriaceae* that were isolated from diarrhoeic calves in Egypt. From 120 calves, 149 isolates of bacteria were recovered, identified, and screened phenotypically against 12 antimicrobials, and molecularly for the presence of the resistance determinants of integrons, ß-lactamases and florfenicol. The findings revealed that 24.8% of the isolates exhibited multidrug resistance. *Escherichia coli* was found to be the most prevalent multidrug resistant species. Class 1 integrons, *bla*_TEM_, and *flo*R genes were detected at incidence rates of 18.8%, 24.8%, and 1.3%, respectively, whereas class 2 integrons and *bla*_CTX-M_ were not detected in any isolates. The higher incidence of the antimicrobial resistance genes indicate the importance of regular monitoring of the antibiotic susceptibilities of isolated bacteria to minimise the risk of human exposure to pathogens that are resistant to antimicrobials.

**Abstract:**

The present study was designed to investigate the presence of genes that conferred resistance to antimicrobials among *Enterobacteriaceae* that were isolated from diarrhoeic calves. A total of 120 faecal samples were collected from diarrhoeic calves that were raised in Kafr El-Sheikh governorate, Egypt. The samples were screened for *Enterobacteriaceae*. A total of 149 isolates of bacteria were recovered and identified; *Escherichia coli* was found to be the most overwhelming species, followed by *Citrobacter diversus*, *Shigella* spp., *Serratia* spp., *Providencia* spp., *Enterobacter* spp., *Klebsiella pneumoniae*, *Proteus* spp., *Klebsiella oxytoca*, and *Morganella morganii*. All isolates were tested for susceptibility to 12 antimicrobials; resistant and intermediately resistant strains were screened by conventional polymerase chain reaction for the presence of antimicrobial resistance genes. Of the 149 isolates, 37 (24.8%) exhibited multidrug resistant phenotypes. The most prevalent multidrug resistant species were *E. coli*, *C. diversus*, *Serratia* spp., *K. pneumoniae*, *Shigella* spp., *Providencia* spp., and *K. oxytoca*. Class 1 integrons were detected in 28 (18.8%) isolates. All isolates were negative for class 2 integrons. The *bla*_TEM_ gene was identified in 37 (24.8%) isolates, whereas no isolates carried the *bla*_CTX-M_ gene_._ The florfenicol gene (*flo*R) was detected in two bacterial isolates (1.3%). The findings of this study reveal that calves may act as potential reservoirs of multidrug resistant bacteria that can be easily transmitted to humans.

## 1. Introduction

Neonatal diarrhoea in young, pre-weaned calves remains one of the main causes of the animals’ morbidity and mortality, and it causes major economic losses in many dairy and beef herds [1]. Antibiotic therapy is used widely in animal medicine to prevent and treat several bacterial infections, including calf diarrhoea [2]. However, the indiscriminate use of antibiotics is associated with evolution of antimicrobial resistance in bacterial pathogens [3].The emergence of antimicrobial resistance among pathogens is a growing concern in veterinary medicine. These resistant organisms pose a threat, not only to animals, but also possibly to humans [4]. Gram-negative bacteria that include members of the family *Enterobacteriaceae* are medically important infectious agents that are present in large numbers in animal guts and are believed to be closely associated with antibiotic resistance [5].

Increasing drug resistance in bacteria is mainly due to mobile genetic elements, such as plasmids, transposons, and integrons, which can be spread easily through bacterial populations [6]. Integrons are the genetic elements most able to capture individual antibiotic resistance genes and, in the process, promote their transcription and expression [7,8]. They are widely disseminated in antibiotic-resistant, clinical isolates of Gram-negative bacteria, and their presence limits the options that are available to treat infectious diseases in humans and animals [9]. To date, nine classes of integrons have been identified; however, integron classes 1 and 2 are the most common in Gram-negative bacteria [10].

Penicillin derivatives (β-lactams) are broad-spectrum, antibacterial agents that are widely used in human and veterinary medicine. Resistance to ampicillin in bacteria is mediated primarily by β-lactamases. Many different β-lactamases have been described, but TEM-, SHV-, OXA-, CMY-, and CTX-M-type β-lactamases are the most predominant [11].

For many years, chloramphenicol was considered the ideal drug to treat salmonellosis in humans and animals. Resistance to chloramphenicol is known to be mediated by plasmid-located enzymes called chloramphenicol acetyltransferases (CAT) [12]. Florfenicol is related to chloramphenicol and shows a similar spectrum of activity, except that it is active at lower concentrations than chloramphenicol against different bacterial isolates [13]. Several studies were published worldwide in recent years that document the bacterial causes of calf diarrhoea as well as antibacterial susceptibility patterns of isolated bacteria [14,15,16,17,18]. However, few publications address the issue of the molecular basis of antimicrobial resistance in bacteria that have been isolated from diarrhoeic calves. Therefore, the objective of the current study was to assess the phenotypes and prevalence of antimicrobial resistance genes in *Enterobacteriaceae* that had been isolated from diarrhoeic calves in Egypt. This assessment would include integrons, ß-lactamases, and florfenicol.

## 2. Materials and Methods

### 2.1. Ethical Considerations

Ethical approval was obtained from the research, publication, and ethics committee of the Faculty of Veterinary Medicine, Kafrelsheikh University, Egypt, which complies with all relevant Egyptian legislation. The ethical approval number is KFS 2017/3.

### 2.2. Sampling, Isolation, and Identification Procedures

A total of 120 faecal swabs were collected from untreated diarrhoeic calves (from one day old up to six months old) that had been raised in raised in three farms including Sakha Mehallet Mousa and Al Karada farms, Kafr El-Sheikh governorate, northern Egypt. Faecal samples were taken using sterile rectal swabs after digital stimulation of the rectal mucosa. Each swab was immersed in a small, sterile, plastic tube that contained 5 mL of sterile normal saline. Then the tube was tightly closed, labelled, and submitted immediately in an ice bag to the bacteriology laboratory of the Faculty of Veterinary Medicine, Kafrelsheikh University, Egypt, where it was cultured for *Enterobacteriaceae*. Briefly, about 1 mL from each sample was inoculated into 9 mL of nutrient broth. After aerobic incubation at 37 °C for 24–48 h, a loopful of the cultivated nutrient broth was streaked onto the surface of the MacConkey agar (Oxoid, Hampshire, UK). After incubation at 37 °C, colonies that showed the morphological characteristics of members of the *Enterobacteriaceae* family were placed into individual nutrient agar slants as pure culture for further identification. Films were prepared from the isolated colonies, stained with Gram′s stain and examined microscopically. The isolates thus obtained were identified by conventional techniques [19]. All bacterial isolates were also confirmed biochemically through use of the API 20E system (bioMérieux, Marcy-l’Étoile, France).

### 2.3. Antimicrobial Susceptibility Testing

Bacterial isolates were tested in vitro for their susceptibility to 12 antimicrobial agents (Oxoid, Hampshire, UK). This was performed through use of a Kirby–Bauer disc diffusion assay, according to the standards and interpretive criteria described by the Clinical and Laboratory Standards Institute (CLSI) [20]. The following antimicrobial agents were tested: ampicillin (AMP), 10 µg; amoxicillin–clavulanic acid (AMC), 30 µg; cefotaxime (CTX), 30 µg; chloramphenicol (CHL), 30 µg; ciprofloxacin (CIP), 30 µg; gentamicin (GEN), 10 µg; nalidixic acid (NAL), 30 µg; norfloxacin (NOR), 10 µg; sulfamethoxazole–trimethoprim (SXT), 25 µg; streptomycin (STR), 10 µg; spectinomycin (SPT), 100 µg; and tetracycline (TET), 30 µg. Susceptibility of the isolates to antimicrobial agents was categorised (as susceptible, intermediate or resistant) by measurement of the inhibition zone, according to interpretive criteria that adhered to the CLSI guidelines. The isolates that displayed resistance to ≥ two different antimicrobial classes were categorised as multidrug resistant.

### 2.4. DNA Extraction and Screening of Antimicrobial Resistance Genes

Genomic DNA of bacterial isolates was extracted through boiling methods. Briefly, a smooth, single colony was inoculated in 5 mL of nutrient broth and incubated at 37 °C for 12 h, and then 200 µL of overnight culture was mixed with 800 µL of distilled water and boiled for 10 min in a heat block. After boiling, the tubes were immediately placed on ice for 5 min followed by centrifugation at 14,000 rpm for 5 min. The supernatant, which contained bacterial DNA, was transferred to a new tube and stored at −20 °C [21]. All isolates were tested more than once for the presence of genes that were resistant to integrons (class1 and 2), ß-lactamases (*bla*_CTX-M_ and *bla*_TEM_), and florfenicol (*flo*R). Primer sequences, target genes and polymerase chain reaction (PCR) products are summarised in Table 1.

Several PCR protocols were used to detect the target genes of the isolates (Table 2).The PCR products were loaded onto 1.0% agarose gel (Sigma-Aldrich Co., St. Louis, MO, USA) that was stained with 0.5 µg/mL ethidium bromide (Sigma-Aldrich Co., St. Louis, MO, USA). The amplified DNAs were electrophoresed at 100 V for 60 min on a mini, horizontal electrophoresis unit (Bio-Rad, Hercules, CA, USA). The gel was then visualised and photographed under an ultraviolet transilluminator.

### 2.5. DNA Sequencing

DNA sequencing of class I integrons was carried out on purified PCR amplicons (QIAquick Gel Extraction Kit, Qiagen Inc., Tokyo, Japan) by application of an ABI automated DNA sequencer (Model 373; Perkin-Elmer, Waltham, MA, USA). DNA strands of the PCR product were sequenced by the dideoxy chain-termination method [25] through use of an ABI automated DNA sequencer (Model 373; Perkin–Elmer, Waltham, MA, USA). The primers 5’CS and 3’CS, which amplify the region between the 5’conserved segment (CS) and the 3’CS of class 1 integrons, were used to sequence from each end of the amplicons as previously described [24]. The sequences were compared with those that are held in GenBank through use of the basic local alignment search tool (BLAST) (http://www.ncbi.nih.gov, accessed on 20 March 2021)

## 3. Results

### 3.1. Occurrence of Multidrug Resistant Enterobacteriaceae in Diarrhoeic Calves

From 120 faecal samples that were analysed from calves with diarrhoea, 149 bacterial isolates were recovered. Based on cultural, morphological, and biochemical characteristics, 10 different types of bacteria were isolated and identified (Table 3). *Escherichia coli* (*E. coli*) was the predominant species (40 isolates; 26.8%), followed by *Citrobacter diversus* (*C. diversus*) (27 isolates; 18%), *Shigella* spp. (24 isolates; 16%), *Serratia* spp. (18 isolates; 12%), *Providencia* spp. (nine isolates; 6%), *Enterobacter* spp. (nine isolates; 6%), *Klebsiella pneumoniae* (*K. pneumoniae*) (nine isolates; 6%), *Proteus* spp. (six isolates; 4%), *Klebsiella oxytoca* (*K. oxytoca*) (four isolates; 2.5%,) and *Morganella morganii* (*M. morganii*) (three isolates; 2%).

The results of antimicrobial disc diffusion tests for the identified bacterial isolates showed that 37 out of the 149 (24.8%) isolates contained multidrug resistant (MDR) phenotypes. As shown in Table 3, *E. coli* was found to be the predominant MDR species (13 isolates; 8.7%), followed by *C. diversus* (eight isolates; 5.4%), *Serratia* spp. (five isolates; 3.3%), *K. pneumoniae* (four isolates; 2.7%), *Shigella* spp. (four isolates; 2.7%), *Providencia* spp. (two isolates; 1.3%) and *K. oxytoca* (one isolate; 0.7%). However, none of the isolates of *Enterobacter*, *M. morganii*, or *Proteus* spp. were found to be multidrug resistant. As summarised in Table 4, the highest level of resistance that was determined among the MDR bacteria was against ampicillin and tetracycline (36 isolates; 97.3% each), followed by amoxicillin–clavulanic acid (34 isolates; 91.9%), streptomycin (32 isolates; 86.5%), sulfamethoxazole–trimethoprim (30 isolates; 81%), nalidixic acid (28 isolates; 75.5%), spectinomycin (24 isolates; 64.9%), gentamicin (23 isolates; 62.2%), ciprofloxacin (nine isolates; 24.3%), chloramphenicol (seven isolates; 18.9%), cefotaxime (six isolates; 16.2%), and norfloxacin (four isolates; 10.8%).

### 3.2. Occurrence of Class 1 and Class 2 Integrons

**Figure 1:**
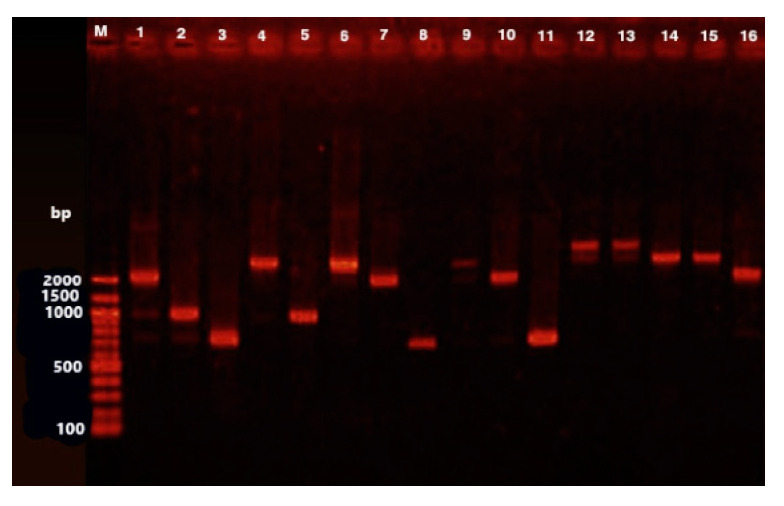
Electrophoresis pattern on agarose gel (1.0%) due to amplicons generated with 5’CS-3’CS primers of class I integrons. Lane M is a 100 bp ladder as a molecular size standard. Lanes 1 to 16 represent gene groups of 0.7 kbp, 1.0 kbp, 1.6 kbp, 2.0 kbp, and 3.0 kbp amplicons of class 1 integrons.

Using 5’CS and 3’CS primers, 0.7 kbp, 1.0 kbp, 1.6 kbp, 2.0 kbp, and 3.0 kbp of amplicons of class 1 integrons were amplified and identified in 28 (18.8%) bacterial isolates (Table 5, Figure 1). The isolates that harboured class 1 integrons were: *E. coli* (11 isolates); *C. diversus* (six isolates); *K. pneumoniae* (four isolates); *Shigella* spp. (four isolates); *Providencia* spp. (two isolates); and *K. oxytoca* (one isolate). DNA sequencing of class 1 integrons identified five different gene cassettes as follows: dihydrofolate reductase types *dfrA1* and *dfrA17*, which confer resistance to trimethoprim; aminoglycoside adenylyltransferase types *aadA1*, *aadA2*, and *aadA5*, which confer resistance to streptomycin and spectinomycin; chloramphenicol acetyltransferase (*catB3*), which confers resistance to chloramphenicol; streptothricin acetyltransferase (*sat1*), which confers resistance to streptothricin; and the β-lactamase gene (*bla*_Pse1_), which confers resistance to ampicillin. It was of note that all isolates were negative for class 2 integrons.

### 3.3. Occurrence of β-Lactamases and Florfenicol Resistance Genes

**Figure 2:**
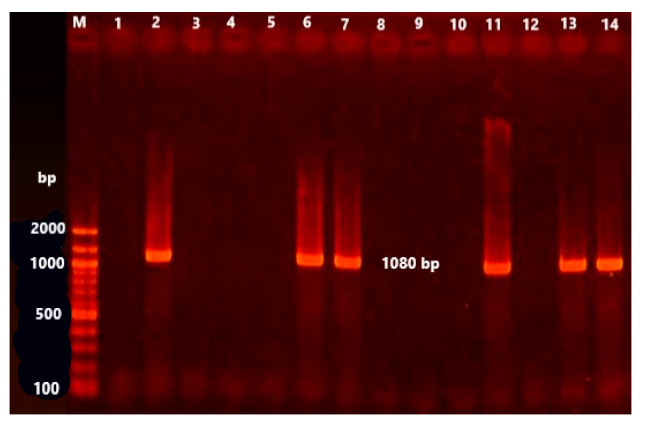
Electrophoresis pattern on agarose gel (1.0%) made by the amplicons for the *bla*_TEM_ genes (1080 bp). Lane M is a 100 bp ladder as a molecular size standard. Lanes 2, 6–7, 11, 13, and 14: positive isolates. Lanes 1, 3–5, 8–10, 12: negative isolates.

**Figure 3:**
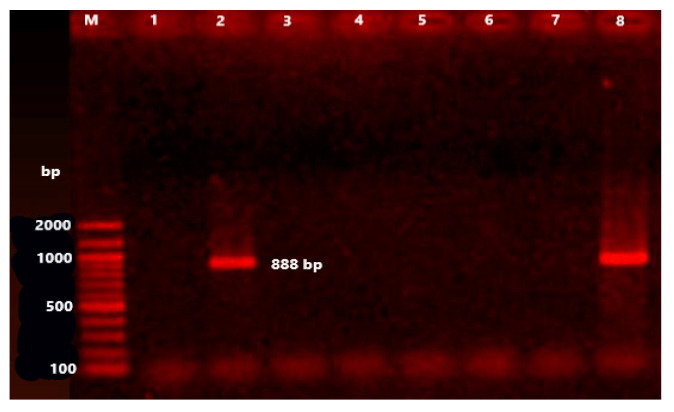
Electrophoresis pattern on agarose gel (1.0%) caused by the amplicons for the *flo*R gene (888 bp). Lane M is a 100 bp ladder as a molecular size standard. Lanes 2, 8: positive isolates. Lanes 1, 3–7: negative isolates.

The *bla*_TEM_ is a narrow-spectrum, β-lactamase gene, which confers resistance against penicillins and first-generation cephalosporins. It was screened in all isolates. PCR and DNA-sequencing identified *bla*_TEM_ in 37 (24.8%) bacterial isolates. The most common isolates that harboured *bla*_TEM_ were: *E. coli* (13 isolates); *C. diversus* (eight isolates); *Serratia* spp. (five isolates); *K. pneumoniae* (four isolates); *Shigella* spp. (four isolates); *Providencia* spp. (two isolates); and *K. oxytoca* (one isolate) (Table 5, Figure 2). It was of note that all isolates were negative for the *bla*_CTX-M_ resistance gene. The florfenicol resistance gene, *flo*R, which confers resistance to chloramphenicol and florfenicol, was identified in two bacterial isolates (1.3%) of *E. coli* and *K. pneumoniae* (Table 5, Figure 3).

## 4. Discussion

In the last few years, considerable attention has been paid to the emergence of antimicrobial resistance among animal pathogens worldwide. This resistance can pose a serious risk regarding the transmission of resistant strains to humans and the environment [1]. In the current study, 149 isolates of *Enterobacteriaceae* were identified from calves with diarrhoea and examined for selected antimicrobial resistance genes. Our findings revealed that 37 (24.8%) isolates showed MDR to two or more antimicrobial agents. The phenotypic characterisation of the tested isolates against 12 antimicrobial agents showed that the isolates exhibited high resistance to ampicillin, tetracycline, amoxicillin–clavulanic acid, streptomycin, sulfamethoxazole–trimethoprim, and nalidixic acid.

Resistant phenotypes of bacteria that have been isolated from diarrhoeic calves have been described in many countries. However, the resistance pattern of the isolated bacteria varies among countries. For example, a study that was carried out by Raska et al. [26] reported that almost 100% of Gram-negative bacteria that were isolated from calves were resistant to ampicillin, tetracycline, and sulphonamides. The greatest *E. coli* resistance to antimicrobials tetracycline and ampicillin was recorded in India [27] and Iran [28]. Moreover, a recent study conducted among human patients in India stated that 82.9%, 54.1%, and 51.9% of bacterial strains that had been isolated from diarrhoeic patients were resistant to ampicillin, tetracycline, and cotrimoxazole, respectively [29]. Our data support a finding that has been reported by earlier studies, which indicates that bacteria of animal origin are commonly resistant to tetracycline, penicillins, and sulphonamides [30]. One possible explanation for the higher resistance of the recovered isolates to these groups of antibiotics (penicillin and tetracyclines) is that they are the most widely used antibiotics for the prevention and treatment of calf diarrhoea in conventional dairies, because they are relatively cheap, can be given orally, and have relatively few side effects.

MDR is defined as the propensity of a cell to exhibit resistance to a wide variety of structurally and functionally unrelated molecules [31]. In the current study, *E. coli* was found to be the predominant multidrug resistant species (13 isolates; 8.7%). This frequency of resistance is similar to that reported in Egypt (10.4%) [32] and in China (4.9%) [33], but lower than the frequencies recorded in Sweden (61%) [34], India (84%) [1], and Egypt (64.3%) [35]. A similar level of MDR among Gram-negative bacteria isolated from cattle with mastitis was reported in Egypt [36]. Such variations in antibiotic resistance in various countries may reflect differences in antimicrobial treatments that are used.

Integrons are considered to contain the most genetic elements that are responsible for dissemination of antimicrobial resistance among bacteria, especially Gram-negative bacteria [9]. In the present investigation, class 1 integrons were detected in 28 (18.8%) of the bacterial isolates. Class 1 integrons that harbour similar gene cassettes to those contained in these groups have been reported in *Salmonella enterica* that have been isolated from humans and animals in the UK [37] and from MDR *Salmonella* isolates from diarrhoeic calves in Egypt [21]. Class 1 integrons were reported in *E. coli* strains that were isolated from diarrhoeic calves in Egypt [32] and China [38] at incidence rates of 10.4% and 59%, respectively. Class 2 integrons are of similar structure to that of class 1 integrons, but they are associated with transposon Tn7 [39]. In this study, all isolates were negative for class 2 integrons. Similar observations were reported previously in Egypt [32] and Uruguay [2].

In this study, *bla*_TEM-1_ was detected in all MDR isolates, which represented 24.8% of the recovered bacteria. However, all examined isolates were negative for the *bla*_CTX-M_ resistance gene. Previous studies declared higher percentages of β-lactamase resistance in Gram-negative bacteria that were isolated from diarrhoeic calves: for example, *Klebsiella* spp. (48.4%) [40], *E. coli* (100%) [32] and *Salmonella* spp. (55.5%) [21]. However, a recent investigation carried out in Egypt described the discovery of *bla*_TEM-1_ in 71.4% of *E. coli* isolated from calves with diarrhoea [35]. Meanwhile, CMY-,CTX-M-, OXA-, SHV-, and TEM-β-lactamases were detected previously in *E. coli* and *Salmonella* spp. that were isolated from diarrhoeic neonatal calves in Egypt [21].

In the current study, the florfenicol resistance gene, *flo*R, was identified only in two (0.013%) bacterial isolates: *E. coli* and *K. pneumoniae*. This finding was identical to that of earlier studies that were performed in Egypt [32] and France [41]. Moreover, the *flo*R gene has been detected previously in Gram-negative bacteria that were isolated from cattle with mastitis in Egypt [36], *E. coli* isolated from neonatal diarrhoeic calves [21] and *E. coli* isolated from cattle in France [41].

## 5. Conclusions

Antimicrobial resistance in bacteria is a serious global health challenge due to the indiscriminate use of antimicrobials in the treatment of infectious diseases in animals. The findings of this study reveal that calves may act as potential reservoirs for multidrug resistant bacteria that can be transmitted easily to humans. The finding of increased incidence of antimicrobial-resistant genes indicates the importance of regular monitoring of the antibiotic susceptibilities of isolated bacteria to minimise the risk of human exposure to pathogens that are resistant to antimicrobials.

## Figures and Tables

**Table 1:** Primer names, target genes, oligonucleotide sequences, and the product sizes used in PCR and DNA sequencing.

Primers	Sequence (5’ to 3’)	Amplicon Size (bp)	Target	References
Integrons				
5’-CS	GGCATCCAAGCAGCAAG	Variable	Class 1 integron	[22]
3’-CS	AAGCAGACTTGACCTGA			
hep74	CGGGATCCCGGACGGCATGCACGATTTGTA	Variable	Class 2 integron	[23]
hep51	GATGCCATCGCAAGTACGAG			
β-Lactamases				
TEM-F	ATAAAATTCTTGAAGACGAAA	1080	*bla*_TEM_	[23]
TEM-R	GACAGTTACCAATGCTTAATC			
CTX-M-F	CGCTTTGCGATGTGCAG	550	*bla*_CTX-M_	[23]
CTX-M-R	ACCGCGATATCGTTGGT			
Florfenicol				
StCM-F	CACGTTGAGCCTCTATATGG	888	*flo*R	[24]
StCM-R	ATGCAGAAGTAGAACGCGAC			

**Table 2:** PCR amplification and DNA sequencing.

Target gene	Primary Denaturation	Amplification (30 Cycles)			Final Extension
		Secondary Denaturation	Annealing	Extension	
Class 1 integron	94 °C, 10 min	95 °C, 60 s	55 °C, 60 s	72 °C, 3 min	72 °C, 10 min
Class 2 integron	94 °C, 10 min	94 °C, 60 s	55 °C, 60 s	72 °C, 3 min	72 °C, 10 min
*bla*_CTX-M_	95 °C, 10 min	95 °C, 30 s	50 °C, 30 s	72 °C, 30 s	72 °C, 10 min
*bla*_TEM_	94 °C, 10 min	94 °C, 30 s	50 °C, 30 s	72 °C, 60 s	72 °C, 10 min
*flo*R	94 °C, 10 min	94 °C, 30 s	50 °C, 30 s	72 °C, 60 s	72 °C, 10 min

**Table 3:** Occurrence of multidrug resistant *Enterobacteriaceae* isolated from diarrhoeic calves.

Bacteria	Recovered isolates		Multidrug resistant isolates	
	%	No.	%	No.
*Escherichia coli*	26.8	40	8.7	13
*Citrobacter diversus*	18	27	5.4	8
*Shigella* spp.	16	24	2.7	4
*Serratia* spp.	12	18	3.3	5
*Providencia* spp.	6	9	1.3	2
*Enterobacter* spp.	6	9	0	-
*Klebsiella pneumoniae*	6	9	2.7	4
*Proteus* spp.	4	6	0	-
*Klebsiella oxytoca*	2.7	4	0.7	1
*Morganella morganii*	2	3	0	-
Total	100	149	24.8	37

**Table 4:** Results of antimicrobial susceptibility testing for multidrug resistance of *Enterobacteriaceae* that were isolated from diarrhoeic calves.

Number of Resistant Isolates (37)								
Class and Antimicrobials	*E. coli* (*n* = 13)	*C. diversus* (*n* = 8)	*Shigella* spp. (*n* = 4)	*Serratia* spp. (*n* = 5)	*Providencia* spp. (*n* = 2)	*K. pneumoniae* (*n* = 4)	*K. oxytoca* (*n* = 1)	Overall Resistance
Penicillins								
Ampicillin	13	8	4	4	2	4	1	36 (97.3%)
Amoxicillin	12	7	3	5	2	4	1	34 (91.9%)
Quinolones and fluoroquinolone								
Nalidixic acid	10	3	3	5	2	4	1	28 (75.5%)
Ciprofloxacin	4	3	0	1	0	0	1	9 (24.3%)
Norfloxacin	3	0	0	1	0	0	0	4 (10.8%)
Aminoglycosides								
Streptomycin	13	6	2	5	2	4	0	32 (86.5%)
Gentamicin	7	4	2	4	1	4	1	23 (62.2%)
Spectinomycin	9	3	3	3	2	4	0	24 (64.9%)
Sulphonamides								
Sulfamethoxazole –trimethoprim	13	5	2	3	2	4	1	30 (81%)
Cephalosporins								
Cefotaxime	4	1	1	0	0	0	0	6 (16.2%)
Phenicols								
Chloramphenicol	3	1	0	0	0	3	0	7 (24.3%)
Tetracyclines								
Tetracycline	13	8	3	5	2	4	1	36 (97.3%)

**Table 5:** Incidence of antimicrobial-resistant genes in multidrug resistant bacteria isolated from diarrhoeic calves.

**No.**	**Bacteria**	**Resistance Phenotype**	**Class1 Integrons**	**Other gene(s)**
1	*E. coli*	AMC, AMP, CIP, GEN, NAL, NOR, SPT, STR, SXT, TET	*dfrA17-aadA5*	*bla*_TEM_
2	*Serratia* spp.	AMC, AMP, GEN, NAL, STR, SXT, TET	*-*	*bla*_TEM_
3	*Shigella* spp.	AMC, AMP, NAL, SPT, STR, SXT, TET	*dfrA17-aadA5*	*bla*_TEM_
4	*Serratia* spp.	AMC, GEN, NAL, NOR, SPT, STR, TET	-	*bla*_TEM_
5	*Serratia* spp.	AMC, AMP, CIP, NAL, SPT, STR, SXT, TET	-	*bla*_TEM_
6	*Serratia* spp.	AMC, AMP, GEN, NAL, SPT, STR, TET	-	*bla*_TEM_
7	*E. coli*	AMC, AMP, CHL, CIP, CTX, GEN, NAL, NOR, SPT, STR, SXT, TET	*dfrA12-orf-aadA2*	*bla*_TEM_
8	*E. coli*	AMC, AMP, GEN, NAL, SPT, STR, SXT, TET	*dfrA1-aadA1*	*bla*_TEM_
9	*Shigella* spp.	AMC, AMP, GEN, STR, TET	*dfrA17-aadA5*	*bla*_TEM_
10	*C. diversus*	AMC, AMP, GEN, STR, TET	*dfrA12-orf aadA2*	*bla*_TEM_
11	*E. coli*	AMC, AMP, STR, SXT, TET	*dfrA17-aadA5*	*bla*_TEM_
12	*C. diversus*	AMC, AMP, NAL, STR, TET	*dfrA17-aadA5*	*bla*_TEM_
13	*E. coli*	AMC, AMP, GEN, NAL, SPT, STR, SXT, TET	*dfrA17-aadA5*	*bla*_TEM_
14	*K. pneumoniae*	AMC, AMP, CHL, GEN, NAL, SPT, STR, SXT, TET	*dfrA12-orf aadA2*	*bla*_TEM_, *flo*R
15	*C. diversus*	AMP, CIP, SPT, STR, TET	*dfrA12-orf aadA2*	*bla*_TEM_
16	*K. pneumoniae*	AMC, AMP, CHL, GEN, NAL, SPT, STR, SXT, TET	*dfrA12-orf aadA2*	*bla*_TEM_
17	*E. coli*	AMC, AMP, CHL, CTX, NAL, SPT, STR, SXT, TET	*dfrA15*	*bla*_TEM_, *flo*R
18	*C. diversus*	AMC, AMP, CHL, GEN, SPT, SXT, TET	*dfrA1-aadA1*	*bla*_TEM_
19	*C. diversus*	AMC, AMP, SPT STR, SXT, TET	*aac(3)-Id-aadA7*	*bla*_TEM_
20	*E.coli*	AMC, AMP, NAL, STR, SXT, TET	*dfrA1-aadA1*	*bla*_TEM_
21	*Shigella* spp.	AMC, AMP, NAL, SPT, SXT, TET	*dfrA12-orf aadA2*	*bla*_TEM_
22	*E. coli*	AMC, AMP, CIP, NAL, NOR, STR, SXT, TET	*dfrA12-orf aadA2*	*bla*_TEM_
23	*E. coli*	AMC, AMP, GEN, SPT, STR, SXT, TET	*aac(3)-Id-aadA7*	*bla*_TEM_
24	*Providencia* spp.	AMC, AMP, NAL, SPT, STR, SXT, TET	*aac(3)-Id-aadA7*	*bla*_TEM_
25	*C. diversus*	AMC, AMP, CIP, GEN, NAL, SXT, TET	*dfrA1-aadA1*	*bla*_TEM_
26	*C. diversus*	AMC, AMP, CTX, NAL, STR, SXT, TET	*-*	*bla*_TEM_
27	*C. diversus*	AMC, AMP, CIP, GEN, STR, SXT, TET	*-*	*bla*_TEM_
28	*Shigella* spp.	AMP, CTX, GEN, NAL, SPT	*dfrA1-aadA1*	*bla*_TEM_
29	*E. coli*	AMC, AMP, CIP, GEN, SPT, STR, SXT, TET	*dfrA17-aadA5*	*bla*_TEM_
30	*E. coli*	AMP, CTX, NAL, SPT, STR, SXT, TET	*dfrA17-aadA5*	*bla*_TEM_
31	*Providencia* spp.	AMC, AMP, GEN, NAL, SPT, STR, SXT, TET	*aac(3)-Id-aadA7*	*bla*_TEM_
32	*E. coli*	AMC, AMP, CHL, NAL, SPT, STR, SXT, TET	*-*	*bla*_TEM_
33	*E. coli*	AMC, AMP, CTX, GEN, NAL, STR, SXT, TET	*-*	*bla*_TEM_
34	*Serratia* spp.	AMC, AMP, GEN, NAL, STR, SXT, TET	*-*	*bla*_TEM_
35	*K. oxytoca*	AMC, AMP, CIP, GEN, NAL, SXT, TET	*dfrA17-aadA5*	*bla*_TEM_
36	*K. pneumoniae*	AMC, AMP, GEN, NAL, SPT, STR, SXT, TET	*dfrA12-orf-aadA2*	*bla*_TEM_
37	*K. pneumoniae*	AMC, AMP, CHL, GEN, NAL, SPT, STR, SXT, TET	*dfrA1-aadA1*	*bla*_TEM_

AMP, ampicillin; AMC, amoxicillin; CHL, chloramphenicol; CIP, ciprofloxacin; CTX, cefotaxime; GEN, gentamicin; NAL, nalidixic acid; NOR, norfloxacin; SPT, spectinomycin; STR, streptomycin; SXT, sulfamethoxazole–trimethoprim; TET, tetracycline.

## Data Availability

The authors confirm that the data that support the findings of this study are available within the article.
